# Enterovirus D68 virus-like particles expressed in *Pichia pastoris* potently induce neutralizing antibody responses and confer protection against lethal viral infection in mice

**DOI:** 10.1038/s41426-017-0005-x

**Published:** 2018-01-10

**Authors:** Chao Zhang, Xueyang Zhang, Wei Zhang, Wenlong Dai, Jing Xie, Liping Ye, Hongli Wang, Huan Chen, Qingwei Liu, Sitang Gong, Lanlan Geng, Zhong Huang

**Affiliations:** 1 Joint Center for Infection and Immunity, Guangzhou Institute of Pediatrics, Department of Gastroenterology, Guangzhou Women and Children’s Medical Center, Guangzhou Medical University, 510623 Guangzhou, China; 2 Unit of Vaccinology & Antiviral Strategies, CAS Key Laboratory of Molecular Virology & Immunology, Institut Pasteur of Shanghai, Chinese Academy of Sciences, 200031 Shanghai, China

## Abstract

Enterovirus D68 (EV-D68) has been increasingly associated with severe respiratory illness and neurological complications in children worldwide. However, no vaccine is currently available to prevent EV-D68 infection. In the present study, we investigated the possibility of developing a virus-like particle (VLP)-based EV-D68 vaccine. We found that co-expression of the P1 precursor and 3CD protease of EV-D68 in *Pichia pastoris* yeast resulted in the generation of EV-D68 VLPs, which were composed of processed VP0, VP1, and VP3 capsid proteins and were visualized as ~30 nm spherical particles. Mice immunized with these VLPs produced serum antibodies capable of specifically neutralizing EV-D68 infections in vitro. The in vivo protective efficacy of the EV-D68 VLP candidate vaccine was assessed in two challenge experiments. The first challenge experiment showed that neonatal mice born to the VLP-immunized dams were fully protected from lethal EV-D68 infection, whereas in the second experiment, passive transfer of anti-VLP sera was found to confer complete protection in the recipient mice. Collectively, these results demonstrate the proof-of-concept for VLP-based broadly effective EV-D68 vaccines.

## Introduction

Enterovirus D68 (EV-D68) is a virus belonging to the *Enterovirus* genus of the *Picornaviridae* family^1^. Like other enteroviruses, the genome of EV-D68 is a positive-sense single-stranded RNA of ~7.4 kb and contains a single open reading frame (ORF) that encodes a large polyprotein^1,2^. This polyprotein can be processed to produce three precursor proteins, P1, P2, and P3. Subsequently, P1 precursor is cleaved by viral protease 3CD to yield three capsid subunit proteins (VP0, VP1, and VP3), all of which co-assemble to form viral capsid shells. After viral RNA encapsidation, VP0 may be further cleaved into VP2 and VP4 via an autocatalytic mechanism^2^. A crystal structure of EV-D68 virus shows that the EV-D68 viral capsid is made of 60 copies of each of VP1, VP2, VP3, and VP4 subunit proteins and it possesses structural features typical for enteroviruses, such as three-fold propeller-like protrusions, star-shaped five-fold plateaus, narrow depressions (canyons) surrounding each plateau, and hydrophobic pockets inside VP1 and directly beneath the canyon floor^3^. Recent studies have identified neuron-specific intercellular adhesion molecule-5 (ICAM-5/telencephalin) and sialic acid as two cellular receptors for EV-D68^4–6^.

EV-D68 was first identified in the United States of America (USA) in 1962^7^, and was once considered to be a rare cause of respiratory disease, with only 26 cases reported between 1970 and 2005 in the USA^8^. However, in the past 10 years, the incidence of EV-D68 infections has remarkably increased all over the world^9,10^. In particular, a nationwide outbreak of EV-D68 associated with severe respiratory illness occurred in the USA in 2014, resulting in a total of 1153 confirmed cases including at least 14 deaths, and it is the largest and most widespread one of EV-D68 outbreaks ever recorded in the world (https://www.cdc.gov/non-polio-enterovirus/about/ev-d68.html#outbreak). Coinciding with this outbreak, 120 acute flaccid myelitis (AFM) cases were reported in the USA, suggesting that this AFM cluster was likely associated with EV-D68 infection^11^. Indeed, several cohort studies confirmed that there is an association between EV-D68 and AFM^12–14^. Thus, EV-D68 infection can cause not only severe respiratory disease but also neurological damage. Over the past 3 years, a significant number of cases of EV-D68 infection have been reported in different countries in America, Asia, or Europe^15–17^, indicating the continued circulation of EV-D68 around the world. Clearly, EV-D68 has posed a serious threat to children’s health and therefore development of effective EV-D68 vaccines should be prioritized. In addition, based on VP1 gene sequences, EV-D68 is divided into three major clades, designated A, B and C, which are currently circulating worldwide^10^.

Virus-like particles (VLPs), which can be produced in recombinant expression systems and resemble inactivated authentic virions in structure and immunogenicity, constitute an effective platform for vaccine development, as showcased by the successful commercialization of VLP-based human papillomavirus and hepatitis B vaccines^18,19^. Previously, our group has produced VLPs for a number of enteroviruses associated with hand, foot, and mouth disease, including enterovirus 71 (EV-A71), coxsackievirus A16 (CV-A16), and coxsackievirus A6 (CV-A6), and demonstrated their protective efficacies in preclinical studies^20–25^. In this study, we investigated the possibility of developing a VLP-based EV-D68 vaccine. Our results showed that EV-D68 VLP could be produced in *Pichia pastoris* (*P. pastoris*) yeast and this VLP antigen elicited neutralizing antibodies capable of protecting mice against lethal EV-D68 infections, indicating that EV-D68 VLP is a promising vaccine candidate.

## Materials and methods

### Cells and viruses

RD cells (ATCC CCL-136) were grown as described previously^26^. PichiaPink^TM^ yeast strain one (Invitrogen, USA) was grown according to the instructions of the manufacturer. EV-D68 strains US/MO/14-18947 (GenBank ID: KM851225), US/KY/14-18953 (GenBank ID: KM851231), and Fermon (GenBank ID: AY426531) were obtained from ATCC and propagated in RD cells. EV-A71 strain EV-A71/G082 and CV-A16 strain CV-A16/SZ05 were described previously^26,27^. All viruses were titrated for the 50% tissue culture infectious dose (TCID50) using RD cells, according to the Reed–Muench method^28^.

### Proteins, inactivated EV-D68 virus, and antibodies

Recombinant VP0, VP1, and VP3 proteins of EV-D68 strain Fermon were separately expressed in *E. coli* cells using a previously described protocol^29^. Briefly, RNA was extracted from Fermon-infected RD cells. The first strand cDNA was synthesized using M-MLV reverse transcriptase (Promega, USA) and oligo (dT) primers. DNA fragments encoding VP0, VP1, and VP3 were amplified individually from cDNA and then cloned into the expression vector pET28b, resulting in plasmids pET-VP0, pET-VP1, and pET-VP3, respectively. The plasmids were transformed separately into *E. coli* BL21 (DE3) cells for expression. Finally, the His-tagged fusion proteins (VP0, VP1, and VP3) were purified by using Ni^2+^ resins. The anti-VP0, anti-VP1, and anti-VP3 polyclonal antibodies were generated by immunization of BALB/c mice with recombinant EV-D68 VP0, VP1 and VP3 proteins, respectively. β-propiolactone-inactivated EV-D68 virus was prepared from US/MO/14-18947-infected RD cells using protocols identical to those described in a previous study^27^. A polyclonal antibody against inactivated EV-D68 was generated in house from BALB/c mice immunized with inactivated EV-D68 virus.

### Vector construction

The P1 gene of EV-D68 strain US/MO/14-18950 (GenBank ID: KM851228) was optimized according to *P. pastoris* preferred codon, synthesized, and subsequently inserted into the expression vector pPink-HC (Invitrogen, USA), yielding a plasmid named pEV-D68-001. To obtain 3CD gene, RNA was extracted from US/MO/14-18947-infected RD cells, and reverse transcribed into cDNA using M-MLV reverse transcriptase (Promega, USA) and oligo (dT) primers; DNA fragment encoding 3CD was amplified by PCR from the cDNA and then cloned into pPink-HC, to generate plasmid pEV-D68-002. For co-expression of P1 and 3CD, the P1 expression cassette in the plasmid pEV-D68-001 was released by *Bgl*II and *BamH*I digestion, and then inserted into *Bgl*II-linearized plasmid pEV-D68-002, resulting in a plasmid designated pEV-D68-003.

### Yeast transformation and screening

Plasmid pEV-D68-003 was linearized by *Afl*II digestion and then transformed into PichiaPink™ Strain one (Invitrogen, USA) by electroporation. The transformed yeast cells were subsequently plated onto PAD selection plates that lacked adenine and then incubated at 30 °C for 4 days. For screening of high-expression recombinant strains, 40 colonies were randomly selected for small-scale expression experiments, and then subjected to cell lysis as described previously^23^. The resulting clarified cell lysates from individual yeast clones were analyzed for EV-D68 protein expression by ELISA and Western blotting assays as described below.

### ELISA and Western blotting assays

For indirect ELISA, wells of 96-well microplates were coated with 5 µl of yeast lysates plus 45 µl of PBS buffer at 37 °C for 2 h; after three washes with PBST buffer, the plates were incubated with 200 μl/well of blocking buffer (5% milk in PBST) for 1 h at 37 °C; after three washes, 50 μl/well of mouse polyclonal antibody against inactivated EV-D68 (diluted 1:1000 in PBST) was added and incubated at 37 °C for 2 h; after washing three times, the plates were added with HRP-conjugated goat anti-mouse IgG (Sigma, USA) for 1 h at 37 °C. After color development, the absorbance of each well at 450 nm was measured in a microplate reader.

Western blotting was carried out as described previously^30^ with a polyclonal antibody against EV-D68 VP3.

### Preparation of EV-D68 VLPs and control antigen

EV-D68 VLPs were prepared from the selected yeast strain with the highest expression level by using a protocol described in a previous study^23^. An empty vector pPink-HC transformed yeast strain was subjected to the identical procedures to produce the negative control antigen. The purified VLPs and negative control antigen were quantified by performing Bradford assay and then used for immunization experiments.

### Electron microscopy

Purified EV-D68 VLP was negatively stained with 0.5% aqueous uranyl acetate and then examined using an FEI Tecnai G2 Spirit electron microscope operating at 120 kV.

### Mouse immunization

The animal studies were approved by the Institutional Animal Care and Use Committee at the Institut Pasteur of Shanghai.

Prior to immunization, antigens (VLP or the negative control antigen, 1 μg/dose) were adsorbed on aluminum hydroxide adjuvant (Invivogen, USA, 500 μg/dose), to make the experimental vaccines. Groups of six female ICR mice aged 6–8 weeks were immunized intraperitoneally (i.p.) with the vaccine formulations at weeks 0 and 3. Blood samples of individual mice were collected at week 5. The resulting sera were heat-inactivated at 56 °C for 30 min and then stored at −80 °C until use.

### Serum antibody measurement

Total anti-EV-D68 IgG antibodies in immunized mouse sera were determined by performing indirect ELISA. Briefly, the microplates were coated with 50 ng/well of inactivated EV-D68 virus at 4 °C overnight and then blocked with 5% milk diluted in PBST for 1 h at 37 °C; serum samples diluted 1:1000 in PBST plus 1% milk were added and incubated at 37 °C for 2 h; subsequently, the plates were incubated with HRP-conjugated goat anti-mouse IgG (Sigma, USA) for 1 h at 37 °C. After color development, the absorbance of each well at 450 nm was measured in a microplate reader.

### Neutralization assay

Neutralizing antibody titers were determined by a micro-neutralization assay. Briefly, in each well of 96-well plates, 50 µl of two-fold serially diluted serum samples were mixed with 50 µl (100 TCID_50_) of EV-D68 and incubated at 37 °C for 1 h. Next, RD cell suspensions (100 μl, 15,000 cells/well) were added to each well and incubated at 33 °C for 3 days. Then, the cells were observed for cytopathic effect (CPE). The neutralizing titers were read as the highest serum dilutions that could fully inhibit CPE.

### In vivo protection assays

The protective efficacy of the experimental vaccines was assessed by two in vivo assays. In one experiment, the female ICR mice that had received two doses of the experimental vaccines were allowed to mate with naive ICR male mice 3 weeks after the last immunization. One-day-old suckling mice born to immunized dams were inoculated i.p. with 1.2 × 10^5^ TCID_50_ of US/MO/14-18947. The challenged mice were monitored daily for survival rates and clinical scores for 14 days. Clinical scores were graded as follows: 0, healthy; 1, lethargy and reduced mobility; 2, limb weakness; 3, limb paralysis; and 4, death.

In another experiment, groups of naive ICR mice less than one day of age were i.p. injected with 20 µl of pooled anti-VLP sera or control sera, respectively, followed by i.p. inoculation with 1.2 × 10^5^ TCID_50_ of US/MO/14-18947 one day later. The challenged mice were monitored daily for clinical scores and survival rates for 14 days using the same criteria as described above.

### Statistical analysis

All statistical analysis was performed using GraphPad Prism version 5. The EV-D68-specific IgG response and neutralizing titers were analyzed by the two-tailed Student’s *t*-test.

## Results

### Expression and characterization of EV-D68 VLPs in *P. pastoris*

**Fig. 1:**
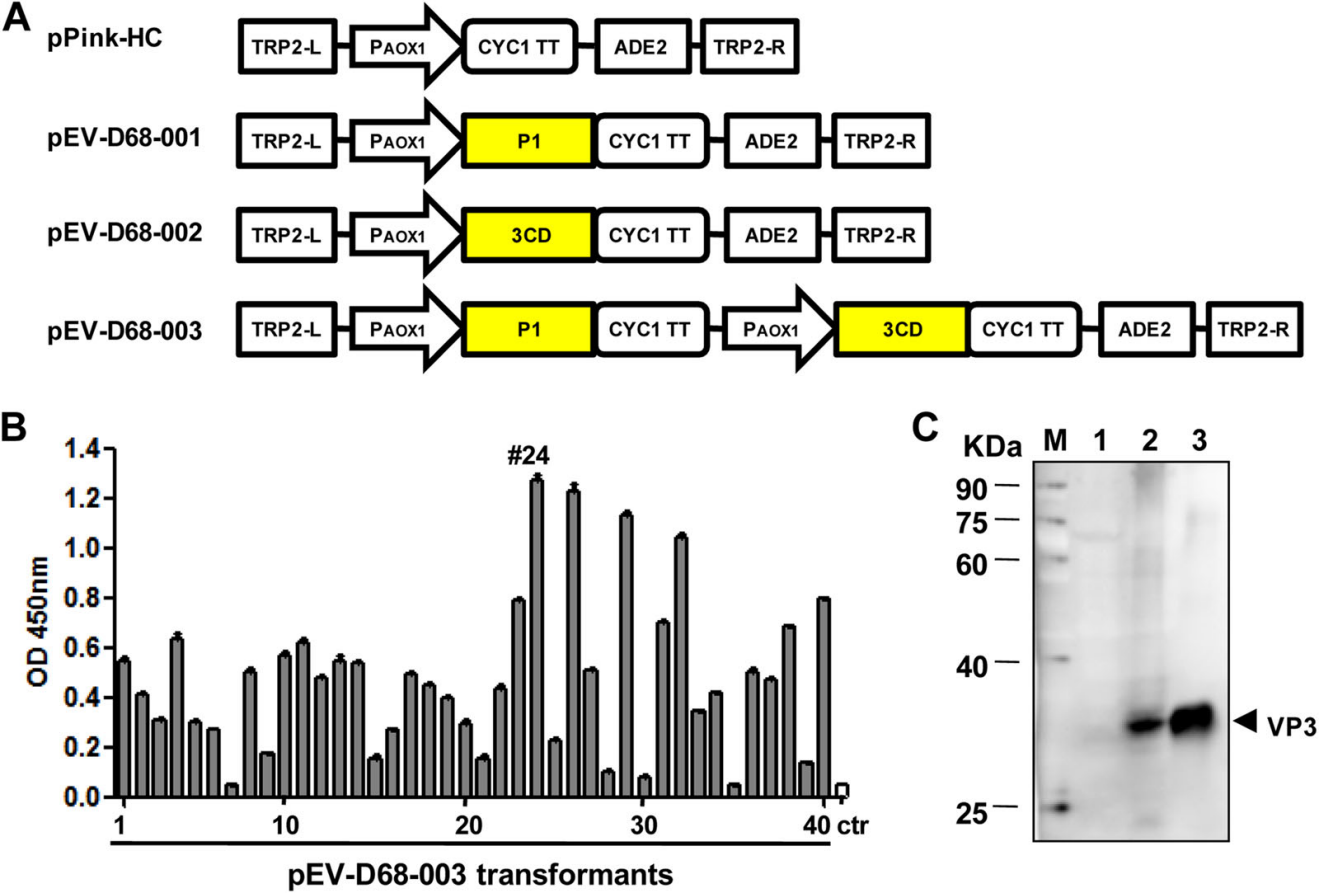
Co-expression of P1 and 3CD of EV-D68 in *P. pastoris* **a** Schematic diagrams of the constructs made in this study. TRP2-L and TRP2-R, the up-stream and down-stream parts of the TRP region, used for homologous recombination; P_AOX1_, AOX1 promoter; CYC1 TT, CYC1 transcription termination region; ADE2, expression cassette encoding phosphoribosylaminoimidazole carboxylase, used as the selection marker. **b** ELISA analysis of cell lysates from pEV-D68-003-transformed yeast clones. A polyclonal antibody against inactivated EV-D68 was used for detection. Cell lysate from a yeast clone transformed with the empty vector pPink-HC was used as the negative control (ctr). Data are mean ± SD of triplicate wells. **c** Western blotting analysis with an anti-VP3 polyclonal antibody. Lane M, marker; lane 1, empty vector-transformed yeast; lane 2, pEV-D68-003-transformed yeast; lane 3, inactivated EV-D68 virus

In order to express EV-D68 VLPs in *P. pastoris*, three constructs were made: pEV-D68-001 and pEV-D68-002 carried only one single P1 and one single 3CD expression cassette, respectively, while pEV-D68-003 contained both cassettes to co-express P1 and 3CD (Fig. 1a). The plasmid pEV-D68-003 was transformed into the PichiaPink^TM^ yeast, and the resulting transformants were analyzed for EV-D68 protein expression by ELISA using a polyclonal antibody against inactivated EV-D68. An empty vector-transformed yeast clone served as the negative control. As shown in Fig. 1b, the pEV-D68-003 transformants showed different levels of expression; clone #24 (designated EV-D68-003-24) exhibited the strongest signal and was chosen for subsequent experiments. To determine whether the P1 precursor was cleaved into subunit proteins by protease 3CD, the transformants were further analyzed by Western blotting with the VP3-specific polyclonal antibody. Purified EV-D68 viron served as the reference standard to mark the position of VP3 protein. As shown in Fig. 1c, the EV-D68-003-24 yeast clone, but not the empty vector-transformed yeast, yielded a specific band at around 30 kDa as did EV-D68 viron. The presence of VP3 protein indicated the successful cleavage of P1 by 3CD.

**Fig. 2:**
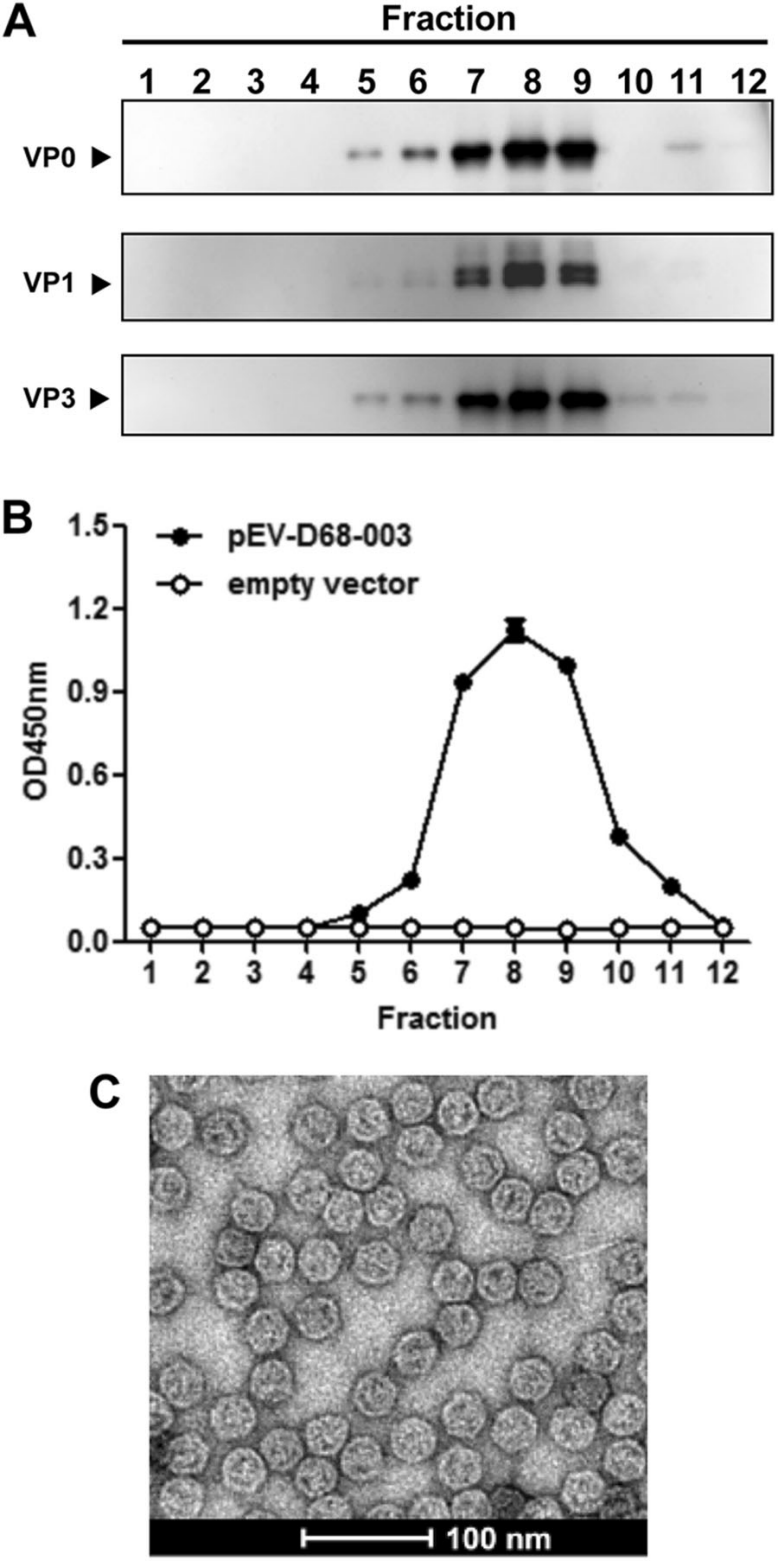
Characterization of EV-D68 VLP assembly **a–b** Sucrose gradient analyses. Lysates from pEV-D68-003-transformed yeast were subjected to 10–50% sucrose gradient sedimentation. Twelve fractions were collected from top to the bottom and analyzed by (**a**) Western blotting with anti-VP0, anti-VP1, or anti-VP3 polyclonal antibodies, respectively, and (**b**) ELISA with a polyclonal antibody against inactivated EV-D68. **c** Electron microscopy of EV-D68 VLPs. Bar = 100 nm

To test whether the resulting EV-D68 proteins assembled into VLPs, sucrose gradient centrifugation of yeast lysates was performed and the resultant gradient fractions were analyzed by Western blotting with VP0-specific, VP1-specific, and VP3-specific antibodies. As shown in Fig. 2a, the VP0, VP1, and VP3 proteins of the expected sizes were observed to co-sediment largely in the factions #7, #8, and #9, implying that the three subunit proteins co-assembled into EV-D68 VLPs. Furthermore, ELISA analysis with EV-D68 virus-specific antibody showed that strong reactivity was observed in the factions #7, #8, and #9 of the lysates from the EV-D68-003-24 yeast clone (Fig. 2b), consistent with the results of Western blotting (Fig. 2a). In contrast, the control yeast sample did not show any reactivity toward the detection antibody (Fig. 2b). Finally, electron microscopy of the peak fractions of the EV-D68-003-24 yeast clone revealed that EV-D68 VLPs were spherical and of diameter ∼30 nm (Fig. 2c). The above results demonstrated the successful expression and assembly of EV-D68 VLPs in *P. pastoris*.

### Neutralizing antibody responses elicited by EV-D68 VLP immunization

**Fig. 3:**
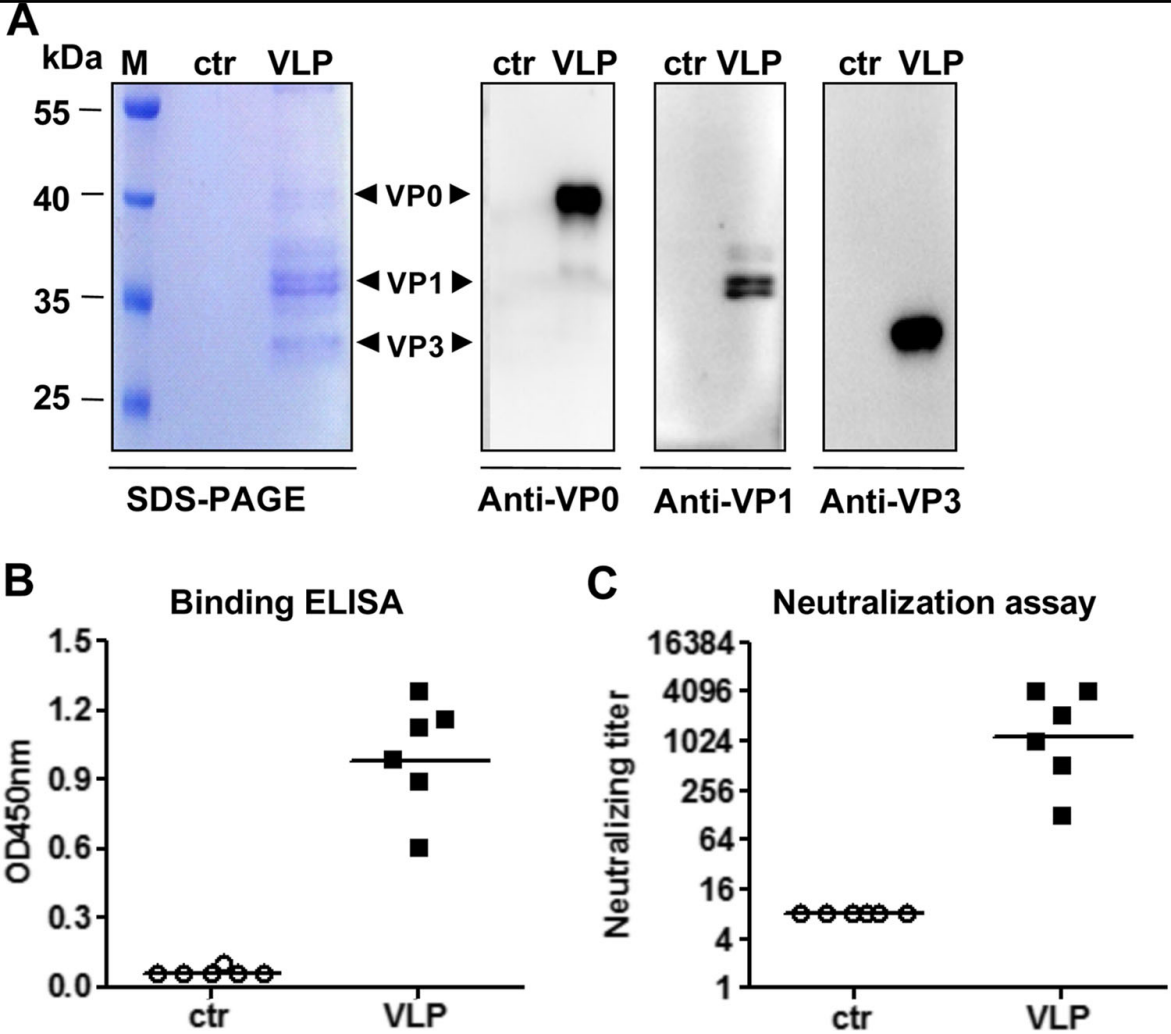
EV-D68 VLPs-induced potent neutralizing antibody responses in mice **a** SDS-PAGE and Western blotting analysis of the purified antigens. Lane M, protein marker; ctr, the control antigen prepared from empty vector-transformed yeast; VLP, purified EV-D68 VLPs. **b** ELISA reactivity of antisera with EV-D68 virus coated on the plate. Mouse antisera taken at 2 weeks after the last immunization were diluted 1:1000 and then used in this assay. **c** Neutralizing antibody titers against US/MO/14-18947 measured by the micro-neutralization assay. The antisera from mice immunized with control antigen did not exhibit any neutralization effect at 1:16 dilution (the lowest dilution tested) and were therefore denoted as a titer of eight for geometric mean titer (GMT) computation. Each symbol represents an individual mouse, and the solid line indicates the GMT of the group

For animal immunization studies, EV-D68 VLPs and the control antigen were prepared with the identical purification procedures from the EV-D68-003-24 yeast clone and the empty vector-transformed yeast cells, respectively. SDS-PAGE and Western blotting analysis revealed that EV-D68 VLPs consisted of VP0, VP1, and VP3 proteins; in contrast, the control antigen did not yield any clearly visible protein band (Fig. 3a). To determine the immunogenicity of the purified antigens, two groups of female ICR mice were immunized twice by i.p. injection of EV-D68 VLPs or the control antigen at 3-week intervals. Antisera obtained 2 weeks after the final immunization were tested for EV-D68-specific serum IgG levels by ELISA using EV-D68 virus as coating antigen. As shown in Fig. 3b, the antisera of the control group did not show any virus-binding activity, whereas all VLP-immunized mouse sera strongly reacted with the capture antigen, indicating that EV-D68 VLPs, but not the control antigen, could elicit virus-specific antibody responses in mice.

We then determined the ability of individual antisera to neutralize live EV-D68 in vitro. As shown in Fig. 3c, the antisera from mice immunized with control antigen did not exhibit any neutralization effect at the lowest dilution tested (1:16) and were therefore denoted as a titer of eight for geometric mean titer (GMT) computation; in contrast, all sera of the VLP group potently neutralized EV-D68 clinical strain US/MO/14-18947 with a geometric mean titer (GMT) of 1149.

Pooled antisera for each group were further tested for their capacity to cross-neutralize heterologous strains and viruses. The VLP-immunized sera strongly neutralized the heterologous EV-D68 strain US/KY/14-18953 and weakly neutralized EV-D68 prototype strain Fermon, but did not display any cross-neutralizing activities against other enteroviruses tested, including EV-A71/G082 and CV-A16/SZ05, at a 1:16 dilution (Table 1). These results indicate that the neutralizing antibodies induced by VLPs were EV-D68-specific.

**Table 1:** Cross-neutralization activity of the pooled antisera against heterologous strains and viruses

Pooled antisera against	Neutralization titer against				
	EV-D68/Fermon	EV-D68/US/MO/14-18947	EV-D68/US/KY/14-18953	EV-A71/G082	CV-A16/SZ05
Control antigen	<16	<16	<16	<16	<16
VLP	32	4096	1024	<16	<16

The lowest serum dilution tested is 1:16

### Maternal immunization with VLPs protected suckling mice against lethal infection

**Fig. 4:**
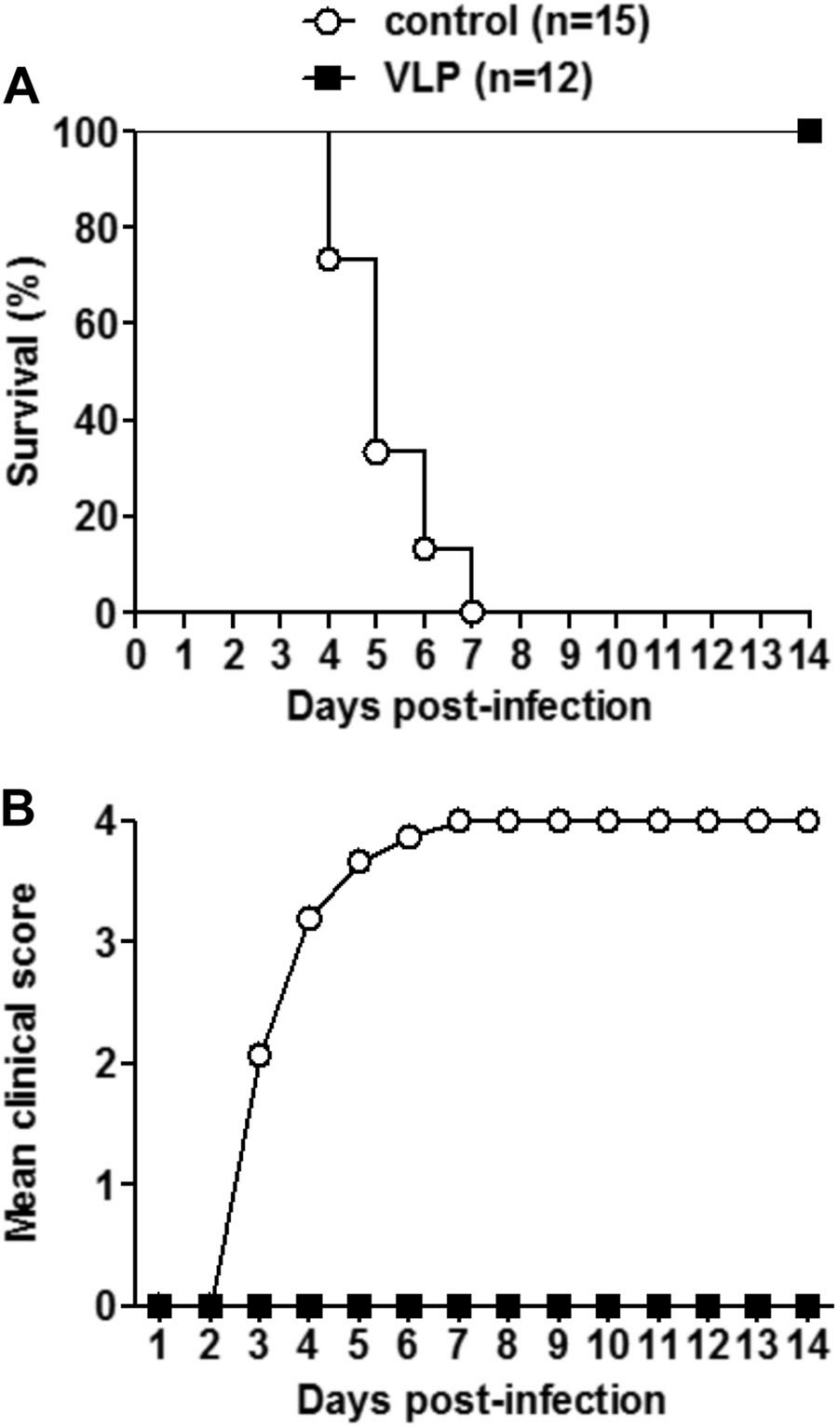
Maternal immunization with EV-D68 VLPs provided complete protection for neonatal mice against lethal challenge One-day-old ICR mice born to dams immunized with EV-D68 VLPs or the control antigen were i.p. injected with 1.2 × 10^5^ TCID_50_ of US/MO/14-18947. All infected mice were observed daily for **a** survival and **b** clinical score for 14 days. Clinical scores were graded as follows: 0, healthy; 1, lethargy and reduced mobility; 2, limb weakness; 3, limb paralysis; 4, death. The numbers of mice in each group were shown in brackets

The in vivo protective efficacy of the EV-D68 VLP vaccine was assessed using a suckling mouse model based on the EV-D68 strain US/MO/14-18947. The immunized female ICR mice were allowed to mate with naive male mice and one-day-old offspring born to immunized dams were inoculated i.p. with a lethal dose of US/MO/14-18947. All suckling mice were observed daily for survival and clinical signs after infection. The pups born from dams immunized with the control antigen gradually show symptoms, such as limb weakness and paralysis, and all of them eventually died 4–7 days post-infection (dpi); in contrast, all suckling mice born to the VLP-immunized dams remained healthy throughout the course (Fig. 4). These results indicate that maternal immunization with EV-D68 VLPs can provide effective protection against lethal challenge in neonatal mice.

### Passive transfer of anti-VLP sera protected suckling mice against lethal infection

**Fig. 5:**
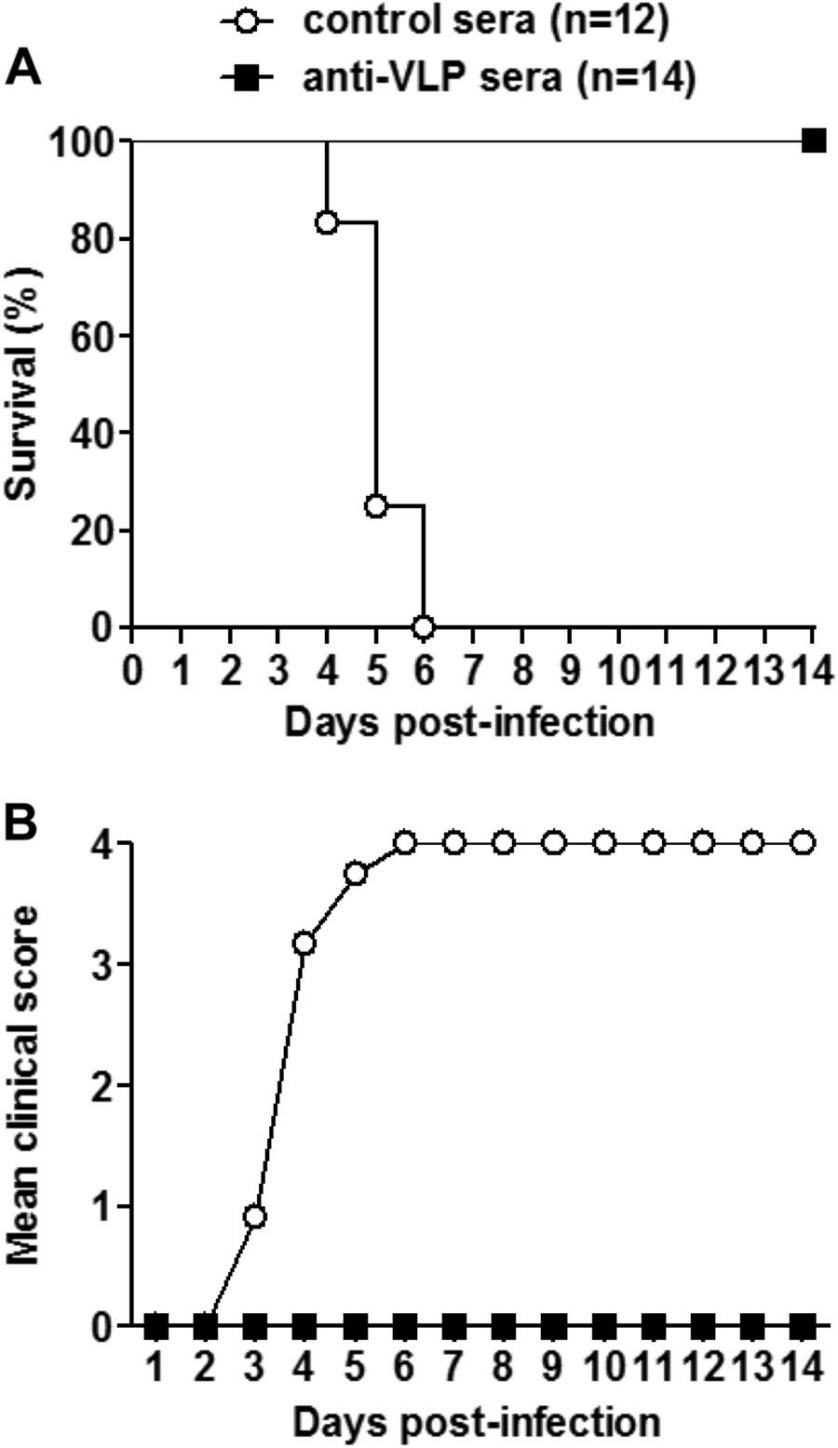
Passive transfer of anti-VLP sera fully protected newborn mice against lethal infection Groups of naive ICR mice (age <24 h) were i.p. injected with 20 µl of anti-VLP or control sera, and 1 day later i.p. inoculated with 1.2 × 10^5^ TCID_50_ of US/MO/14-18947. Following challenge, mice were monitored daily for **a** survival and **b** clinical score for 14 days. Clinical scores were graded as described in the legend of Fig. 4. The numbers of mice in each group were shown in brackets

We also evaluated the in vivo protective ability of anti-VLP sera against lethal EV-D68 infection. Groups of naive ICR mice less than one day of age were administrated with anti-VLP or control sera, and 24 h later challenged with US/MO/14-18947. As shown in Fig. 5, the mice receiving the control sera began to develop disease symptoms at 3 dpi, and all died by 6 dpi; whereas all of the anti-VLP sera treated neonatal mice survived without any clinical signs.

## Discussion

Thus far, no vaccine for EV-D68 is available. In recent years, EV-D68 infections are increasingly associated with severe respiratory illness and neurological complications, underscoring the necessity of developing safe and efficacious vaccines to prevent EV-D68 infections^14^. The present study was aimed to evaluate the possibility of developing a VLP-based novel EV-D68 vaccine. Our results demonstrated for the first time that yeast-produced EV-D68 VLP could induce effective protective immunity in an animal model of EV-D68 infection, therefore representing an elite EV-D68 vaccine candidate.

EV-D68 VLP was generated in *P. pastoris* yeast co-expressing P1 and 3CD proteins of EV-D68. This co-expression and subsequent proteolytic cleavage led to the generation of individual capsid subunit proteins VP0, VP1, and VP3 (Figs. 2a and 3a), all of which co-assemble into VLP as demonstrated by sucrose gradient analysis and electron microscopy (Fig. 2). These data demonstrate that co-expression of P1 and 3CD is an effective approach for producing EV-D68 VLP in *P. pastoris*. Previsouly, we and other research groups have used the same approach to successfully produce VLPs of EV-A71, CV-A16, or CV-A6 in *P. pastoris* ^23–25,31^, and in *Saccharomyces cerevisiae*^32,33^. Together, these results suggest that the VLP expression strategy is likely universal for enteroviuses and should be prioritized in designing and developing recombinant protein-based enteroviral vaccines.

In the present study, EV-D68 VLPs were found to elicit in mice the production of serum antibodies that potently neutralized EV-D68 infection in vitro (Fig. 3 and Table 1). The anti-VLP sera were able to neutralize all three tested EV-D68 strains, but had no neutralization effect on the representative strains of two other enteroviruses, EV-A71 and CV-A16 (Fig. 3 and Table 1). The lack of intertypic cross-neutralization by the anti-EV-D68 VLP sera is as expected, because the amino acid sequence homology in the capsid (P1) region is fairly low between EV-D68 and EV-A71 (~46%) or between EV-D68 and CV-A16 (~47%). It is worth noting that the anti-VLP sera exhibited distinct neutralization potency against the three EV-D68 strains tested (Table 1). Sequence comparison (data not shown) shows that the VLP vaccine strain (US/MO/14-18950, clade B) shares the highest homology in the P1 region with the US/MO/14-18947 strain (clade B, 99.5%), followed by US/KY/14-18953 (clade A, 95.8%), and the prototype strain Fermon (93.3%). Surface loops of individual VP1, VP2, and VP3 capsid subunit proteins are often the antigenic sites for enteroviruses and harbor neutralizing epitopes^34–38^. The alignment (data not shown) reveals that the surface loop regions of the vaccine strain US/MO/14-18950 are 98.4%, 94.2%, and 89.1% identical to those of the US/MO/14-18947, US/KY/14-18953, or Fermon strains, respectively. Clearly, for the anti-VLP sera, their neutralization potency against a given EV-D68 strain correlates with degree of sequence homology between the vaccine strain and the strain being tested. These findings suggest that it is important to continue selecting a single consensus sequence or test multivalent formulations to ensure elicitation of broadly neutralizing antibodies, which is the key to developing a VLP-based universal EV-D68 vaccine.

In the present study, the protective efficacy of the EV-D68 VLP vaccine candidate was assessed in two challenge experiments. In the first experiment, neonatal mice born to the VLP-immunized dams were fully protected from lethal EV-D68 challenge (Fig. 4). In the second experiment, passive transfer of anti-VLP sera conferred complete protection in the recipient mice (Fig. 5). These results not only demonstrated the potent prophylactic efficacy of the VLP vaccine, but also indicated that antibodies, most likely neutralizing antibodies, mediate* in vivo* protection against EV-D68 infection. In addition, we and others have previously demonstrated that recombinant EV-A71 VLP and CV-A16 VLP produced in yeast or baculovirus expression systems were also capable of inducing the production of neutralizing antibodies, which could provide efficient protection to mice against lethal infections with their respective viruses^20, 21, 23, 24, 32,39^. Taken together, these findings suggest that VLP is an effective vaccine strategy for some enteroviruses.

In summary, our study reveals that EV-D68 VLPs can be readily produced in *P. pastoris* and they efficiently elicit neutralizing antibodies able to protect against lethal EV-D68 challenge in mice. These results provide proof-of-concept for the development of a VLP-based universal EV-D68 vaccine.
